# *Nicotiana attenuata* Data Hub (*Na*DH): an integrative platform for exploring genomic, transcriptomic and metabolomic data in wild tobacco

**DOI:** 10.1186/s12864-016-3465-9

**Published:** 2017-01-13

**Authors:** Thomas Brockmöller, Zhihao Ling, Dapeng Li, Emmanuel Gaquerel, Ian T. Baldwin, Shuqing Xu

**Affiliations:** 1 Department of Molecular Ecology, Max Planck Institute for Chemical Ecology, Hans-Knöll-Straße 8, D-07745 Jena, Germany; 2 Centre for Organismal Studies, Heidelberg University, Im Neuenheimer Feld 360, Heidelberg, D-69120 Germany

**Keywords:** *Nicotiana attenuata*, Phylogenomics, Transcriptomics, genomics, Metabolomics, Co-expression analysis

## Abstract

**Background:**

*Nicotiana attenuata* (coyote tobacco) is an ecological model for studying plant-environment interactions and plant gene function under real-world conditions. During the last decade, large amounts of genomic, transcriptomic and metabolomic data have been generated with this plant which has provided new insights into how native plants interact with herbivores, pollinators and microbes. However, an integrative and open access platform that allows for the efficient mining of these -omics data remained unavailable until now.

**Description:**

We present the *Nicotiana attenuata* Data Hub (*Na*DH) as a centralized platform for integrating and visualizing genomic, phylogenomic, transcriptomic and metabolomic data in *N. attenuata*. The *Na*DH currently hosts collections of predicted protein coding sequences of 11 plant species, including two recently sequenced *Nicotiana* species, and their functional annotations, 222 microarray datasets from 10 different experiments, a transcriptomic atlas based on 20 RNA-seq expression profiles and a metabolomic atlas based on 895 metabolite spectra analyzed by mass spectrometry. We implemented several visualization tools, including a modified version of the Electronic Fluorescent Pictograph (eFP) browser, co-expression networks and the Interactive Tree Of Life (iTOL) for studying gene expression divergence among duplicated homologous. In addition, the *Na*DH allows researchers to query phylogenetic trees of 16,305 gene families and provides tools for analyzing their evolutionary history. Furthermore, we also implemented tools to identify co-expressed genes and metabolites, which can be used for predicting the functions of genes. Using the transcription factor *NaMYB8* as an example, we illustrate that the tools and data in *Na*DH can facilitate identification of candidate genes involved in the biosynthesis of specialized metabolites.

**Conclusion:**

The *Na*DH provides interactive visualization and data analysis tools that integrate the expression and evolutionary history of genes in *Nicotiana,* which can facilitate rapid gene discovery and comparative genomic analysis. Because *N. attenuata* shares many genome-wide features with other *Nicotiana* species including cultivated tobacco, and hence *Na*DH can be a resource for exploring the function and evolution of genes in *Nicotiana* species in general. The *Na*DH can be accessed at: http://nadh.ice.mpg.de/.

**Electronic supplementary material:**

The online version of this article (doi:10.1186/s12864-016-3465-9) contains supplementary material, which is available to authorized users.

## Background

*Nicotiana attenuata,* is a diploid wild tobacco native to the Great Basin desert of the United States with populations across Utah, Nevada, Arizona, Oregon, and California. This plant has adapted to an ecological niche defined by the post-fire environment, where soils tend to be nitrogen-rich and biotic stresses are highly dynamic [1]. During the last decade, *N. attenuata* has been developed as a model organism to study plant-environment interactions in its native environment [2–6], and a large number of transcriptomic and metabolomic datasets have been generated with this plant. For example, more than 230 transcriptomic data from *N. attenuata* have been submitted to the NCBI GEO database. However, to efficiently analyze, explore and visualize such genome-wide metabolomic and transcriptomic data remain challenging for individual researchers. In particular, most of these data were not centralized and integrated. Recently, we sequenced and annotated the genomes of *N. attenuata* and its close relative *N. obtusifolia* [7], which provided an opportunity to create tools for centralizing, integrating and visualizing these omics data from this plant.

Specialized metabolites are of special importance in the defenses of plants, therefore, understanding their regulation and their evolutionary history are of central interests in plant biology. However, identifying genes involved in the biosynthesis of specialized metabolites remains difficult due to the large number of gene duplication events in plant genomes, and the structural diversity of the metabolites produced by plants. Recently, studies suggest that co-expression analysis is a powerful tool to rapidly identify genes involved in the biosynthesis of specialized metabolites, because many of these genes are often co-expressed [8]. However, such co-expression analysis often involves large amounts of data and remains difficult to handle for researchers who are not familiar with sophisticated statistics and lack programming skills.

**Fig. 1:**
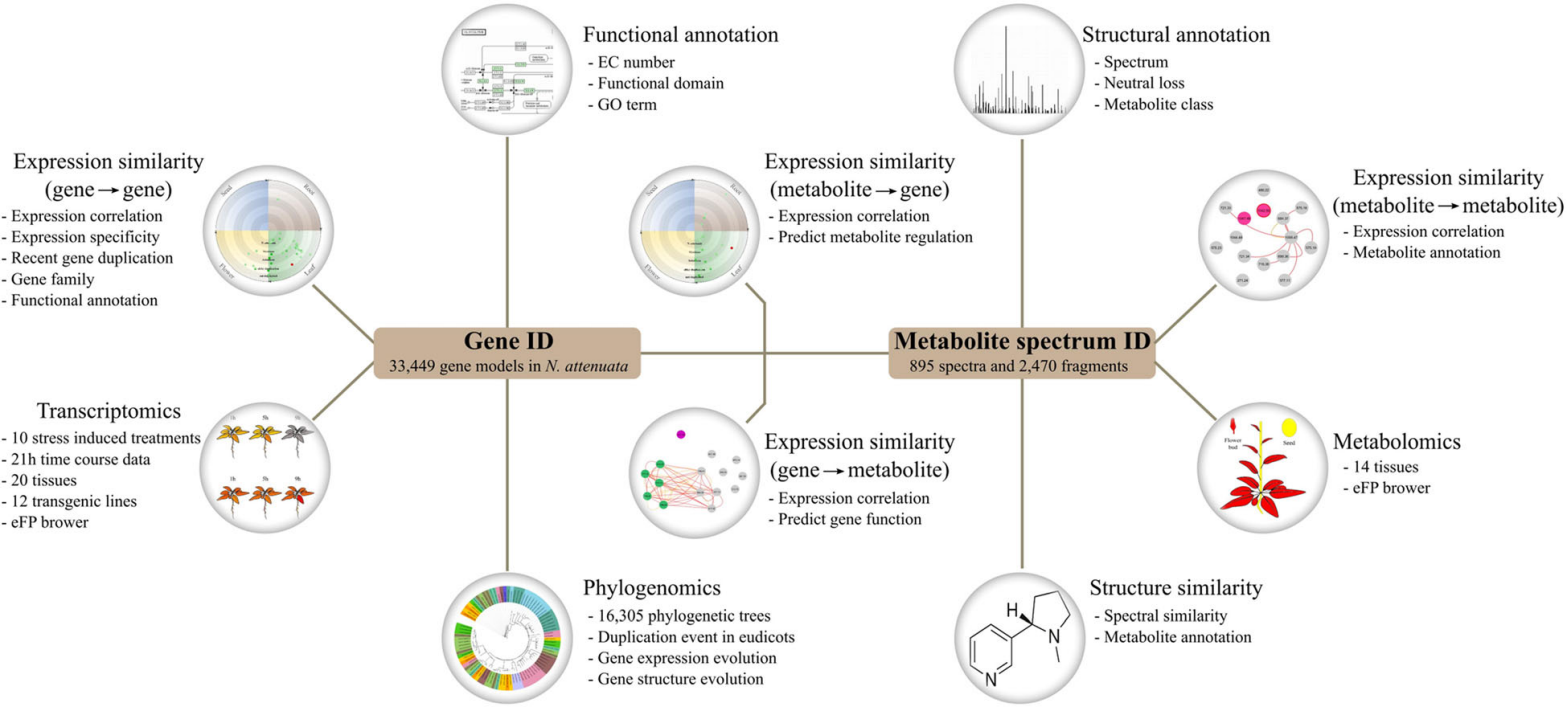
Overview of data structure and utilities in the *Na*DH. The *Na*DH consists of 10 major utilities, which can be accessed by either gene ID or metabolite spectrum ID

Here, we present the *Nicotiana attenuata* Data Hub (*Na*DH, http://nadh.ice.mpg.de), a centralized publicly available platform for storing and integrating genomic, transcriptomic and metabolomic data from *N. attenuata* (Fig. 1). To provide user-friendly data analysis and visualization, we implemented tools from the Electronic Fluorescent Pictograph (eFP) browser, co-expression networks and the Interactive Tree Of Life (iTOL). Using the genes from the biosynthetic pathway of phenolamides as an example, we show that *Na*DH users can rapidly identify genes involved in specialized metabolites and make inferences on their evolution history.

## Construction and content

### Genomic data

The *Na*DH includes 33,449 and 27,911 predicted gene models from *N. attenuata* (release r2.0) and *N. obtusifolia* genomes (release r1.0), respectively. For comparative genomic analysis, additional gene sequences and structures from nine dicot plant genomes are also included in the database (Table 1). To provide functional annotations, the predicted enzyme commission (EC) identities, gene ontology (GO) terms, and protein domains are included.

**Table 1:** Overview of included species for comparative genomics

Species	Version	# of genes	URL	Reference
*N. attenuata*	r2.0	33,449	http://nadh.ice.mpg.de/NaDH/download/	[7]
*N. obtusifolia*	r1.0	27,911	http://nadh.ice.mpg.de/NaDH/download/	[7]
*A. thaliana*	TAIR 10	27,416	http://phytozome.jgi.doe.gov/arabidopsis	[9, 10]
*C. annuum*	v2.0	35,336	http://peppersequence.genomics.cn/page/species/download.jsp	[11]
*C. sativus*	v1.0	21,503	http://phytozome.jgi.doe.gov/cucumber	[12]
*M. guttatus*	v2.0	28,140	http://phytozome.jgi.doe.gov/mimulus	[13]
*P. trichocarpa*	v3.0	41,335	http://phytozome.jgi.doe.gov/poplar	[14]
*S. lycopersicum*	ITAG2.3	34,727	http://phytozome.jgi.doe.gov/tomato	[15]
*S. melongena*	v2.5.1	42,035	ftp://ftp.kazusa.or.jp/pub/eggplant/	[16]
*S. tuberosum*	v3.4	35,119	http://phytozome.jgi.doe.gov/potato	[17]
*V. vinifera*	Genoscope.12X	26,346	http://phytozome.jgi.doe.gov/grape	[18]

### Gene families and phylogeny

The *Na*DH includes 23,340 homologous groups constructed based on protein coding sequences from 11 eudicot species (Table 1). PhyML was used to construct phylogenetic trees with high confidence from these homologous groups that contain more than two genes. In total, 16,305 trees containing 255,404 genes (of which 28,610 are from *N. attenuata*) are included in the *Na*DH. In addition, 81,859 gene duplication events detected from high confidence phylogenetic trees (approximate Bayes branch supports of greater than 0.9 for the target node and its two child nodes) using the species-overlapping algorithm implemented in Notung-2.6 [19, 20] are also included in the database. The majority of gene duplication events in *N. attenuata* are found at the Solanaceae branch (Table 2), consistent with the observation that species of Solanaceae share a whole-genome triplication event.

**Table 2:** The number of detected duplication events in *N. attenuata*

Duplication time	# of duplications
*N. attenuata* specific	3,929
Shared among *Nicotiana spp.*	2,577
Shared among Solanaceae	6,760
Shared with *M. guttatus*	240
Shared among core eudicots	8,548
Total	22,054

A large number of duplication events is shared among all Solanaceae, which supports a shared whole-genome triplication event

### Transcriptomic data

The *Na*DH contains expression profiles from both RNA-seq and microarray datasets [21–23]. For the RNA-seq datasets (Illumina HiSeq 2000, pair-end sequencing, NCBI accession number: PRJNA317743), the expression level (transcript per million, TPM) [24] of each gene from different tissues sampled from leaves, seeds, roots, stems and flowers are included (Table 3). In total, 21,970 genes were expressed in at least one tissue (TPM greater than 5). Roots contain the largest number of expressed genes (Table 3). For the microarray dataset, 222 microarrays (based on Agilent platform: GPL13527) from *N. attenuata* leaves, roots and flowers are included. The probes of this microarray platform were mapped to the *N. attenuata* genome and the uniquely mapped probes were annotated according to gene predictions. In total, this microarray platform contains the expression profiles of 27,374 predicted *N. attenuata* genes. The microarray datasets are organized according to their corresponding experiments and the detailed information on the genotypes, developmental stages, treatments of the plants that provided the samples (Additional file 1) are provided.

**Table 3:** Overview of RNA-Seq data

Library ID/ SRA ID	Tissue	Treatment/development stage	# of expressed genes	Additional note on sampling procedure
NA1498ROT SRX1804895	Root	Rosette stage plants, treated with 5 μL 1:1 diluted *M. sexta* oral secretion three times in leaves	15,499	Roots of rosette stage plants that were treated three times on leaves were collected for RNA extraction. The treatments were performed at 10 am and 6 pm on the day before sampling and 10 am on the day of sampling. Samples were collected at 11 am.
NA1500LET SRX1804896	Leaf	Rosette stage plants, treated with 5 μL 1:1 diluted *M. sexta* oral secretion three times in leaves	12,179	Local leaves of rosette stage plants that were treated three times on leaves were collected for RNA extraction. The treatments were performed at10 am, 6 pm on the day before sampling and 10 am on the day of sampling. Samples were collected at 11 am.
NA1717LEC SRX1804554	Leaf	Rosette stage plants, no treatment	11,840	Rosette stage leaves were collected for RNA extraction. Samples were collected at 11 am.
NA1504STT SRX1804900	Stem	Rosette stage plants, treated with 5 μL 1:1 diluted *M. sexta* oral secretion three times in leaves	14,682	Stems of rosette stage plants that were treated three times on leaves were collected for RNA extraction. The treatments were performed at 10 am, 6 pm on the day before sampling and 10 am on the day of sampling. Samples were collected at 11 am.
NA1505COE SRX1804901	Corolla	Early developmental stage, no treatment	13,662	Samples were collected in the afternoon, 60 samples were pooled.
NA1515COL SRX1804913	Corolla	Late developmental stage, no treatment	13,486	Samples were collected at 6 pm (open flowers) and 9 am (closed flower after opening), 4-10 samples were pooled.
NA1506STI SRX1804902	Stigma	Mature stigma, no treatment	14,485	Stigma samples were collected in the afternoon, 40 samples were pooled.
NA1507POL SRX1804903	Pollen tube	No treatment	3,490	Pollen tubes were pooled.
NA1508SNP SRX1804904	Style	Mature style without pollination	13,492	Styles were collected at 7 am, anthers were removed one day before, and 50 samples were pooled.
NA1509STO SRX1804905	Style	Mature style, pollinated with pollens from different genotype	13,365	Styles were collected at two hours after pollination, at 7 am. Anthers were removed one day before, and 30 samples were pooled.
NA1510STS SRX1804906	Style	Mature style, self-pollinated	13,533	Styles were collected at two hours after pollination, at 7 am. Anthers were removed one day before, and 30 samples were pooled.
NA1511NEC SRX1804907	Nectary	Mature nectary, no treatment	12,928	Samples were collected in the afternoon, 60 samples were pooled.
NA1512ANT SRX1804908	Anther	Mature anther no treatment	11,550	Samples were collected in the afternoon, 60 samples were pooled.
NA1513OVA SRX1804909	Ovary	Mature ovary, no treatment	13,960	Samples were collected in the afternoon, 60 samples were pooled.
NA1514PED SRX1804910	Pedicel	Mature pedicel, no treatment	14,550	Samples collected at 9 am (heading down) and 4 pm (heading up) were pooled.
NA1516OFL SRX1804911	Flower	Fully opened flowers, no treatment	14,390	Both morning (7 am) and evening (6 pm) flowers were collected, 1 sample of each were pooled.
NA1517FLB SRX1804912	Flower bud	Two early developmental stages of flowers, no treatment	14,543	Samples were collected at 6 pm, 1 bud and 1 middle stage flower were collected. Sepals were removed from the samples.
NA1501SES SRX1804897	Seed	Treated with liquid smoke	9,227	100 mg seeds treated with 1:50 diluted liquid smoke solution for 9-15 min were used for RNA extraction.
NA1502SEW SRX1804898	Seed	Treated with water	8,872	100 mg seeds treated with water for 9-15 min were used for total RNA extraction.
NA1503SED SRX1804899	Seed	Dry seeds	8,681	100 mg dried seeds directly used for total RNA extraction.

The raw reads information and methods used for generating these data are available under NCBI accession number PRJNA317743

### Metabolomic data

Metabolomic data from 14 isolated tissues of *N. attenuata* growing under controlled conditions in glasshouse were curated. This includes a pool of all non-senescing rosette leaves, combined lower, middle and higher segments of the stem, the complete root system, dried seeds, complete floral buds of 8 mm length, complete sepal ring, the nectary, the ovary (not including the nectary), the style, anthers, filaments (not including anthers), and the corolla tube and limb, collected at anthesis. Pools of 100 mg tissues were extracted using 80% methanol. Independent extractions were also conducted with 20% methanol. Samples were analyzed using UHPLC-ESI/qTOF-MS in positive ion mode. MS/MS data collection was achieved via a previously-described pipeline [25] and 895 reconstructed MS/MS spectra were obtained [26]. This MS/MS dataset has also been deposited in the EMBL EBI open metabolomics database MetaboLights: www.ebi.ac.uk (accession no. MTBLS335).

### Gene-to-gene co-expression

To facilitate the identification of co-regulated genes in *N. attenuata*, we calculated the pairwise expression correlation co-efficiency based on RNA-seq data from 20 different tissues using three different methods: Gini, Spearman and Pearson [27]. In total, 15,216 informative genes (with TPM greater than 5 in at least one tissue and a variance greater than 1) were used and gene pairs with absolute expression similarity greater than 0.65 were considered for the final dataset. All data are stored in *Na*DH and can be visualized in a network graph.

### Metabolite-to-metabolite co-expression

Metabolite-to-metabolite tissue associations were calculated using Ochiai similarity based on binary metabolite dataset (containing 14 tissue vectors), with a cutoff of 2 on the ZMAD transformed values [28]. The metabolite-to-metabolite pairwise associations were calculated across the dataset by comparing each metabolite spectrum with the other metabolite spectra [26]. All data are stored in the underlying database for fast accessing under the utilities of *Na*DH.

### Gene-to-metabolite co-expression

Gene**-**to-metabolite tissue associations were calculated using Ochiai similarity with binary gene and metabolite data (presence and absence) generated from 12 shared tissues between the transcriptome dataset and the metabolome dataset (a cutoff of transcriptome dataset is ZMAD transformed TPM greater than 3 and a cutoff of metabolome dataset is ZMAD transformed value greater than 2). Gene expression was centered by median and median-absolute-deviation (MAD) to obtain a relative expression level [29]. In total, 23,075 genes and 895 metabolite spectra with expression levels above the threshold were used for the network constructions [26]. The pairwise correlations were calculated using Ochiai correlations based on the transformed binary values [28] and only Ochiai correlation coefficient (occ) greater than 0.3 were considered for the final dataset. The resulting network is based on a correlation matrix between 18,046 genes and 887 metabolite spectra.

### Metabolite structure similarity

Metabolite structure similarity was calculated from pairwise MS/MS alignments based on spectral fragment similarity and common neutral losses similarity (NL). A standard normalized dot product (NDP), also referred to as the cosine correlation method for spectral comparison, was applied for the calculations of spectral fragment similarity. The NL-based similarity between individual MS/MS was implemented using a list of 52 neutral losses (NLs) commonly encountered during tandem MS fragmentation as well as more specific ones that had been previously annotated for MS/MS spectra of *N. attenuata* secondary metabolite classes [25].

### Database architecture and implementation details

An overview of the *Na*DH database architecture is shown in Fig. 1. All data are connected to the gene and metabolite. The data storage function was implemented using the open-source relational management system MySQL to store all data except the genome and gene sequence information. The website was developed using Kohana - an open source, object-oriented model-view-controller (MVC) web framework (https://kohanaframework.org/). The unique filesystem design of the Kohana web framework allows a modular design of all *Na*DH features and enables easy implementations of new functions in the future. The visualization of gene and metabolites expression was implemented by a modified version of the eFP Browser [30]. For interactive visualization of the different co-expression networks, we implemented the open-source graph visualization tool Cytoscape.js (https://github.com/cytoscape/cytoscape.js).

## Utility and discussion

### Search functions

Search functions are implemented for finding genes and metabolites. Genes of *N. attenuata* can be searched by name, functional annotation (InterPro domain, EC number, Gene Ontology identifier), orthologous genes in other species and sequence similarity based on BLAST. Metabolite spectra and fragments can be searched by name, metabolite class and measurements (m/z value, retention time). In both scenarios, a table summarizes information of the corresponding genes or metabolite spectra, and guides users to downstream analysis.

### Gene-to-gene co-expression analysis

**Fig. 2:**
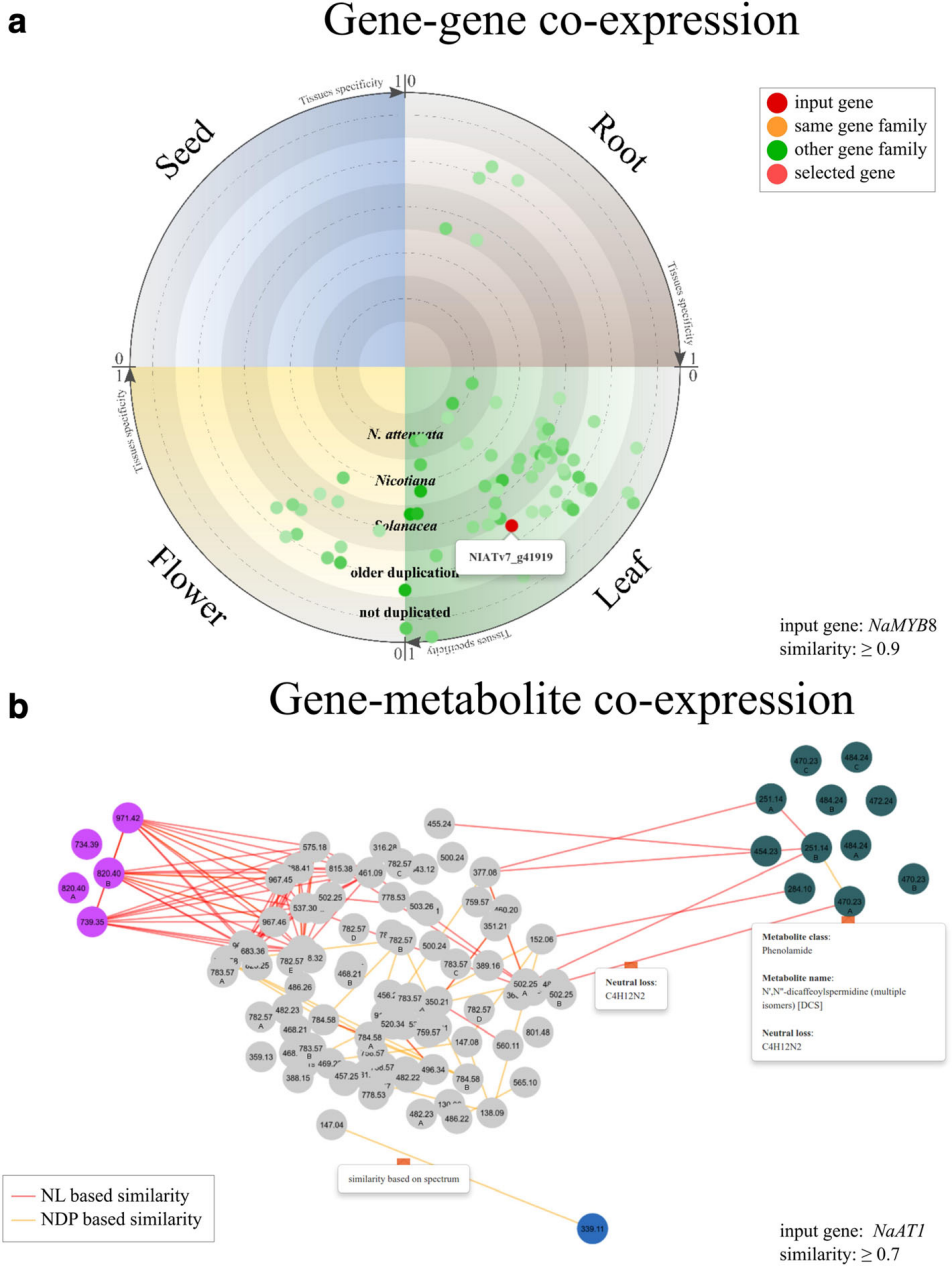
Examples of visualization on co-expressed genes and metabolites. **a** Genes that are co-expressed with a transcription factor (TF) *NaMYB8* (NIATv7_g41919) (*red*). Each node represents a co-expressed gene, radiating position of the node represents the most recent duplication events it has experienced and clock-wise position of the node represents the highest expression among four tissues and relative tissue specificity. **b** Metabolite spectra that are co-expressed with *NaAT1* (NIATv7_g11614), a gene from phenolamides pathway. Color indicate different functional classes of metabolite spectra: *dark green*: phenolamides; *pink*: different 17-hydrogeranyllinalool diterpene glycosides; *blue*: quinate conjugates; *grey*: not annotated metabolite spectra. Node number shows the m/z value of the metabolite spectrum and letters refers to different isotopes. Edges indicate structure similarities among metabolite spectra: *yellow*: NDP based similarity, *red*: NL based similarity. NDP was calculated based on shared fragments and NL was calculated based on shared neutral losses

This function can be used to understand the regulatory mechanisms and predict putative functions of genes. The input is the identifier of a gene, and the outputs are genes co-expressed with the input gene above the user defined threshold (correlation coefficient) and this is presented in both an interactive network graph and table formats. In the resulting co-expression network graph, each node represents a co-expressed gene, radiating position of the node represents the most recent duplication events it has experienced and clock-wise position of the node represents the region with the highest expression among four tissues (leaves, roots, flower buds, and seeds) and relative tissue specificity. The resulting table shows more detailed functional annotation of each node. Figure 2a shows an example output for the transcription factor *NaMYB8* (NIATv7_g41919).

### Metabolite-to-metabolite co-expression analysis

This function can be used to find co-regulated metabolite spectra, which might indicate co-occurrence in biosynthetic pathways and signal cascades. The input of this function is the identifier of a metabolite spectrum of interest, and all co-expressed metabolite spectra above the user-defined threshold and the results are presented in co-expression network graph and table formats. In the co-expression network, each node represents a metabolite spectrum, the color of the nodes represents the annotated class of the corresponding metabolite spectrum, and the edge represents the structural similarity between two nodes: a yellow edge for NDP and red edge for NL. The network can be re-arranged based on expression similarity values or annotated metabolite classes.

### Gene-to-metabolite co-expression analysis

Co-expression between gene and metabolite can be used to both infer putative functions of the genes and to identify candidate biosynthetic pathways of the metabolites [31–34]. In the *Na*DH, we provide a function to find bi-directional searches for co-expressed genes and metabolite spectra. For searching metabolite spectra that are co-expressed with a gene of interest, the input is an identifier of the gene and the output is a metabolite spectra network with each node representing a metabolite spectrum and each edge representing the structural similarity between two metabolite spectra. In order to search for genes that co-express with a metabolite spectrum of interest, the input is an identifier of the metabolite spectrum and the output is the gene co-expression network with each node representing a gene and the position of the node representing the duplication history and expression (similar to gene-gene co-expression network graph). Figure 2b shows the co-expressed metabolite spectra for the gene *NaAT1* (NIATv7_g11614).

### Phylogenetic analysis

**Fig. 3:**
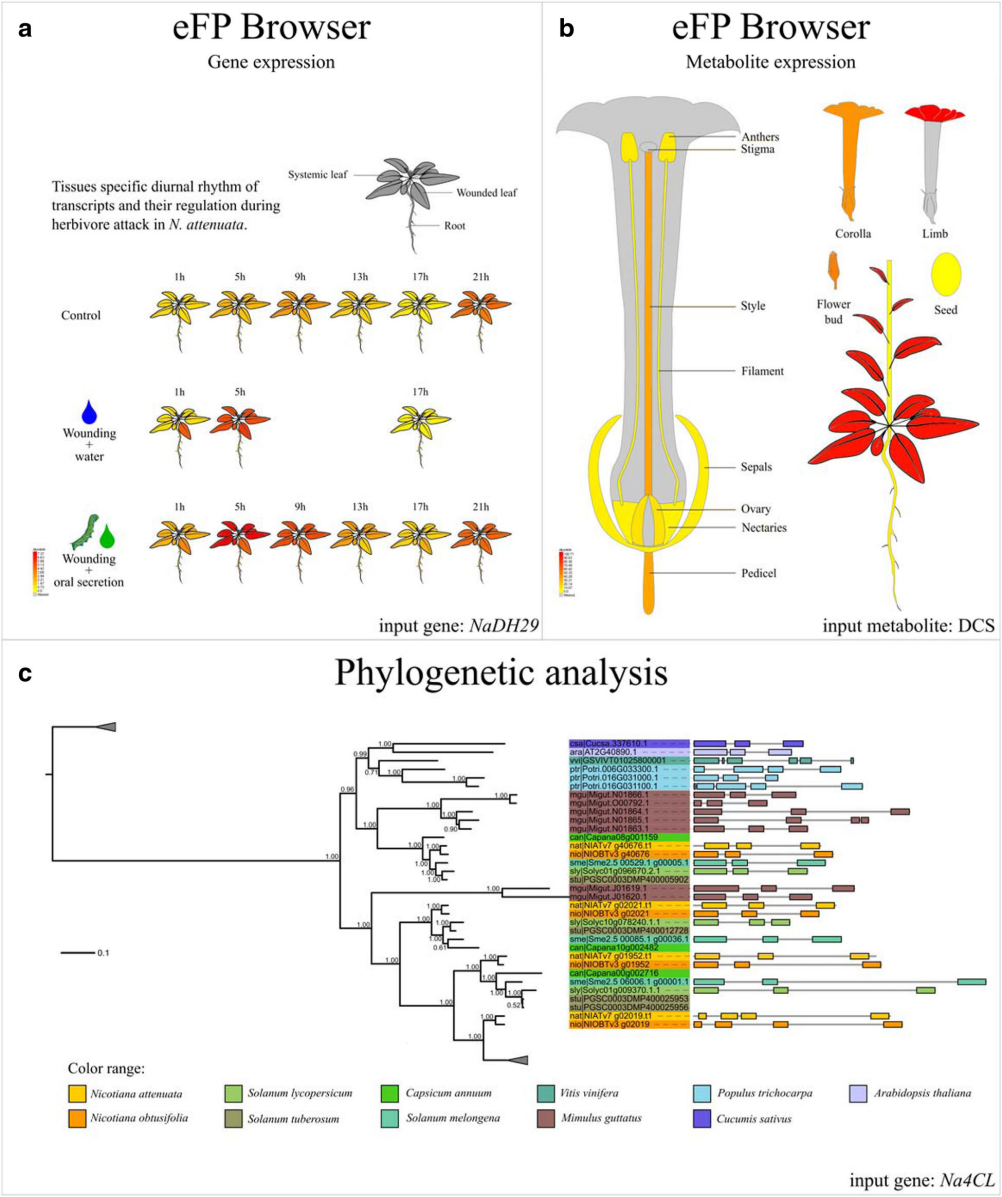
Example of visualizing gene expression and evolution. **a** Expression of *NaDH29* (NIATv7_g06682) in leaves and roots of control or wounding or *M. sexta* oral secretion induced plants. **b** Presence and absence of *N*’,*N*”-dicaffeoylspermidine (DCS) [m/z: 470.2284287, retention time: 395.616992] different *N. attenuata* tissues. **c** Phylogenetic tree of *4CL* gene family visualized using iTOL. Gene structure of each gene is shown on the *right side*. *Colors* refer to different species

In the *Na*DH, a phylogenetic tree can be directly uploaded and visualized with iTOL [35, 36]. The input is an identifier of the gene of interest, and the output is a phylogenetic tree that integrates the expression of *N. attenuata* genes among 20 different tissues. In addition, the intron-exon structures were also integrated with the phylogenetic tree to provide further information on the evolutionary history of the gene. Figure 3c shows an example of the output for the gene DH29 of the phenolamides pathway.

### Expression visualization

The expression of genes and metabolites can be visualized via a modified version of the eFP Browser developed by Nicholas J. Provart et al [30]. The input is either the identifier of a gene (or a probe ID from the microarray) or metabolite spectrum of interest and the output is the expression of the gene (or probe) or precursor of a metabolite spectrum mapped to each tissue or treatment, respectively. The expression levels of the gene are shown as a heatmap with yellow and red colors indicate low and high expression, respectively. The binary expression of the precursor of a metabolite spectrum is shown as a heatmap with red and yellow colors indicate expressed and not expressed, respectively. Figure 3a and b show an example output for a gene and metabolite in the eFP Browser, respectively. The expression values are also provided as a table or bar chart for user-specific analysis. In addition, we also implemented a function to compare and visualize the expression of multiple genes and precursors of metabolite spectra among different tissues.

### Example analysis

The evolution and diversity of specialized metabolites in plants are largely shaped by gene duplication events [37]. Consequently, to find which of the duplicated copies are involved in the biosynthesis and regulation of specific secondary metabolites is challenging. Using the above-described utilities in the *Na*DH and genes known to be involved in phenolamides biosynthetic pathway as an example, we show that the integration of gene-to-gene, gene-to-metabolite, metabolite-to-metabolite and gene duplication history can help to identify genes that are involved in specialized metabolites in the genus *Nicotiana*.

**Fig. 4:**
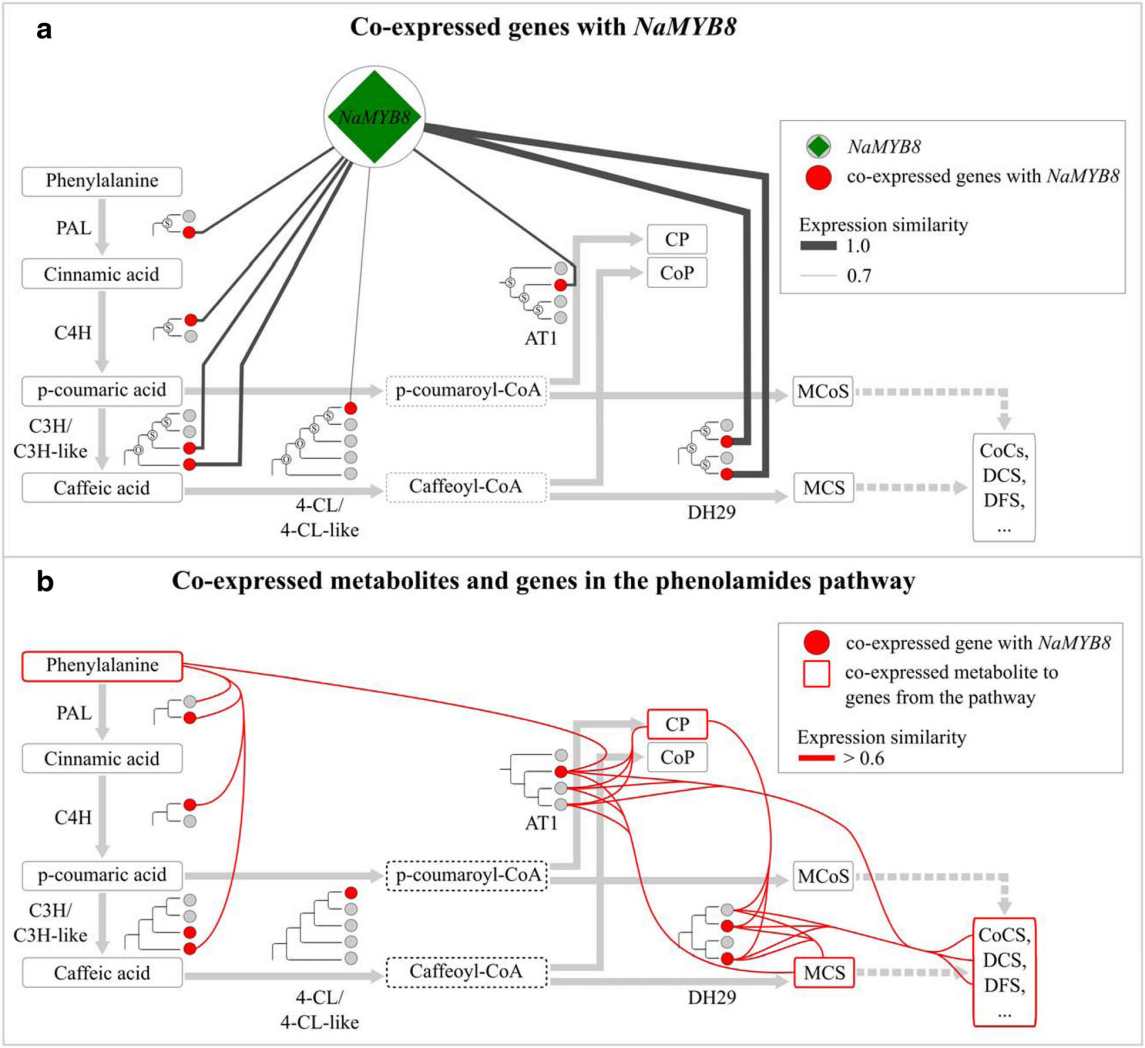
Co-expressed genes and metabolite spectra of the phenolamides pathway. **a** Genes co-expressed with *NaMYB8* (NIATv7_g41919). Each *circle* indicates one gene and the tree structure visualizes their phylogenetic history and the age of the duplication events: N: *Nicotiana* specific; S: Solanaceae specific; O: older duplication. The *red color* indicates that the gene is correlated with *NaMYB8*. The thickness of the *black lines* connecting *NaMYB8* with other genes indicates the expression correlation coefficient. PAL: Phenylalanine ammonia-lyase; C4H: cinnamate-4-hydroxylase; C3H: coumarate-3-hydroxylase; 4CL: 4-coumarate coenzyme A ligase. **b** Co-expression of metabolites and genes from the phenolamides pathway. CP: *N*-caffeoylputrescine, CoP: *N*-coumaroylputrescine, MCoS: *N*-coumarylspermidine, MCS: *N*-caffeoylspermidine, CoCS: *N*-coumaryl, caffeoyl spermidine, DCS: *N’*,*N”*-dicaffeoylspermidine, DFS: *N’*,*N”*-di-feruloyl-spermidine, dashed line: unknown reaction, *dashed box*: metabolite not measurable with used mass spectrometry method. *Red lines* indicate correlation coefficient higher than 0.6

Phenolamides, a group of diverse metabolites abundant in many plant reproductive organs, are rapidly induced after herbivore attack in vegetative tissues of several Solanaceae species and play an important role as induced chemical defenses. The biosynthesis of phenolamides originates from the main phenylpropanoid pathway via *N*-acyltransferase-dependent conjugation to polyamines or aryl monoamines (Fig. 4) [38, 39]. Similar to the biosynthetic pathways of many other secondary metabolites, genes involved in the phenylpropanoid pathway contain multiple copies (Fig. 4). Because several genes involved in the regulation and biosynthesis of phenolamides have been functionally characterized in *N. attenuata*, this group of metabolites provides an ideal example to test the utility of the *Na*DH.

One of the key components that regulates the biosynthesis of phenolamides in *N. attenuata* is the R2R3-MYB transcription factor, *NaMYB8* (NIATv7_g41919) [40]. We first searched for all genes that were co-expressed with *NaMYB8* with a cutoff with a gini correlation coefficient (gcc) greater than 0.7, which resulted in 2,620 co-expressed genes. Among these genes, we searched for homologs that are putatively involved in biosynthetic steps of the main phenylpropanoid pathway. Although in each step, several copies were found in *N. attenuata*, only one or two copies were co-expressed with *NaMYB8*. Among them, functional characterization using virus-induced gene silencing (VIGS) revealed that AT1, CV86 and DH29 are indeed involved in the biosynthesis of herbivore-induced phenolamides, such as caffeoylputrescine (CP) and *N’,N”*-dicaffeoylspermidine (DCS), which function as anti-herbivore chemical defenses [40]. The duplication history of these genes also showed that most of the recent duplications of these genes were from the Solanaceae branch, suggesting the whole genome triplication event of the Solanaceae contributed to the evolution of herbivore-induced phenolamides in *Nicotiana*. Additional co-expression analysis of gene-metabolite and metabolite-metabolite associations showed that the key metabolites (phenylalanine, MCS, CP and DCS) and genes (*NaPAL*, *NaC3H*, *NaDH29*) from the pathway can be retrieved by searching highly co-expressed genes and metabolites (Fig. 4b). Although such co-expression analysis can only be used for the metabolites that are synthesized in the tissues that they accumulate in, these results suggest that using the utilities implemented in the *Na*DH, users can rapidly identify co-expressed genes and metabolites that are involved in the same pathway.

## Conclusion

We present the *Na*DH, which integrates genomic, transcriptomic, and metabolomic data in *N. attenuata* and provides useful tools for the interactive visualization of gene expression divergence and gene duplication history. Additional tools for finding co-expressed genes and metabolites can facilitate rapid gene discovery for specialized metabolites in *N. attenuata* and infer their evolutionary paths. Since the most of genome-wide features are shared among the genus *Nicotiana*, the *Na*DH can also be used to explore the function and evolution of genes in other *Nicotiana* species.

## Availability of data and materials

The datasets generated and/or analysed during the current study are available in N*a*DH (http://nadh.ice.mpg.de/NaDH/).

## Additional file

Additional file 1: Overview of microarray datasets that can be visualized via the eFP browser in *Na*DH. Different unique experiments are included that consist of different genotypes, treatments and time points. EV: empty vector; WT: wild type; W: induced by water; OS: induced by *M. sexta* oral secretion; VIGS: virus-induced gene silencing; GLV: green leaf volatile; VOC: volatile organic compound. (DOCX 17 kb)

## Abbreviations

GLV: Green leaf volatiles
NDP: Normalized dot product
NL: Neutral losses
OS: *M. sexta* oral secretion
TPM: Transcripts per million
VIGS: Virus-induced gene silencing
VOC: Volatile organic compound
WT: Wild type
