# Supplementary material for: Nicotiana attenuata Data Hub (NaDH): an integrative platform for exploring genomic, transcriptomic and metabolomic data in wild tobacco
Source: BMC Genomics. 2017 Jan 13;18:79. doi: 10.1186/s12864-016-3465-9 (PMC5237228; doi:10.1186/s12864-016-3465-9)
Supplement: Additional file 1: — Overview of microarray datasets that can be visualized via the eFP browser in NaDH. Different unique experiments are included that consist of different genotypes, treatments and time points. EV: empty vector; WT: wild type; W: induced by water; OS: induced by M. sexta oral secretion; VIGS: virus-induced gene silencing; GLV: green leaf volatile; VOC: volatile organic compound. (DOCX 17 kb) [file 12864_2016_3465_MOESM1_ESM.docx]

**Additional file 1: Overview of microarray datasets that can be visualized via the eFP browser in *Na*DH.**

| **Dataset ID** | **Genotype used for microarray** | **Treatment** | **Tissue** | **Developmental stage** | **# of arrays** | **Data source** | **Reference** | **Additional notes on the experiment** |
| --- | --- | --- | --- | --- | --- | --- | --- | --- |
| *NaHER1* | WT, *irHER1* | Wounding + OS | Leaves | Rosette stage | 6 | Molecular Ecology Department in MPI-CE | - | Leaves induced with W+OS were used for total RNA extraction. The treatments were performed at 9.30 am and samples were collected at 12.30 pm. |
| Cytokines and senescence | WT, SAG:IPT | *M. sexta* neonates feeding | Young rosette leaves | Early flowering stage | 12 | Molecular Ecology Department in MPI-CE | - | Leaves from the flowering plants that were fed by *M. sexta* for 3 days were used for total RNA extraction. Samples were harvested at 9 am. |
| Coronatine spray on *irAOC* | *irAOC* | H_2_O ethanol spray, 1µM coronatine spray | Corolla, pistil, nectary | Flower buds before opening | 6 | GSE52765 | [38] | Continuous control treatment, treatment happened every other day, Treatment: H_2_O ethanol control spray, 1µM Coronatine spray |
| *NaMYB5* transcription factor | WT, *irMYB5* | Wounding + OS | Leaves | Rosette stage | 6 | Molecular Ecology Department in MPI-CE | - | Leaves induced with W+OS at 9 am were collected at 11 am. |
| *NaMYC2* transcription factors | EV, *MYC2*-VIGS, *MYC2-like* VIGS, *MYC2-MYC2-like* double VIGS | Wounding + OS | Leaves | Rosette stage | 12 | GSE45608 | [39] | Leaves induced with W+OS at 9:30 am and samples were collected 1h later (10:30 am). |
| *N. attenuata* under herbivore attack | WT | Control, wounding, wounding + OS | Leaves and roots | Rosette stage | 138 | GSE30287 | [40] | Leaves were induced at 1pm, treated, leaves, systemic leaves and roots from different time points were collected. |
| Volatile exposure - 6 h | WT, *irLOX2/3*, 35s::*TPS10*, irlox2/3x35S::*TPS10* | VOC exposure for 6 h | Leaves | Rosette stage | 12 | Molecular Ecology Department in MPI-CE | - | Leaves from WT plants that exposed to volatiles from W+OS-treated plants with different genotype were collected for RNA extraction. The samples were exposed for 6h after treatment, which started at 6:17 am. |
| Volatile exposure - 30 min | WT, *irLOX2* | VOC exposure for 30 min and GLV supplementation | Leaves | Rosette stage | 12 | Molecular Ecology Department in MPI-CE | - | Leaves from WT plants that exposed to volatiles from W+OS-treated plants with different genotype were collected for RNA extraction. The samples were exposed for 30 min after treatment, which started at 3:30 pm. |
| *WRKY3/6* | WT, *irWRKY3*, *irWRKY6*, *irWRKY3/6* | *M. sexta* neonates feeding | Leaves | Rosette stage | 12 | Molecular Ecology Department in MPI-CE | - | The experiment started at 14:15 pm by putting 5 *M. sexta* neonates. Every 1h after the last one, 5 more neonates were added and samples were collected after 5h feeding. |
| *WRKY9* | WT, *irWRKY9* | *M. sexta* neonates feeding | Leaves | Rosette stage | 6 | Molecular Ecology Department in MPI-CE | - | The experiment started at 10 am by putting 5 *M. sexta* neonates. Every 1h after the last one, 5 more neonates were added and samples were collected after 5h feeding. |

Different unique experiments are included that consist of different genotypes, treatments and time points. EV: empty vector; WT: wild type; W: induced by water; OS: induced by *M. sexta* oral secretion; VIGS: virus-induced gene silencing; GLV: green leaf volatile; VOC: volatile organic compound.
